# Supplementary material for: Creep-dilatancy development at a transform plate boundary
Source: Nat Commun. 2022 Apr 8;13:1913. doi: 10.1038/s41467-022-29558-0 (PMC8993799; doi:10.1038/s41467-022-29558-0)
Supplement: Supplementary file 1 — Supplementary Information [file 41467_2022_29558_MOESM1_ESM.pdf]

Supplementary Information: Creep-dilatancy development at a transform plate boundary

Nabil Sultan[1*], Shane Murphy[1], Vincent Riboulot[1], Louis Géli[1]

[1] Ifremer, Département REM, Unité des Géosciences Marines, F-29280 Plouzané, France.
[*]Corresponding author.

NS: nabil.sultan@ifremer.fr
SM: shane.murphy@ifremer.fr
VR: vincent.riboulot@ifremer.fr
LG : louis.geli@ifremer.fr

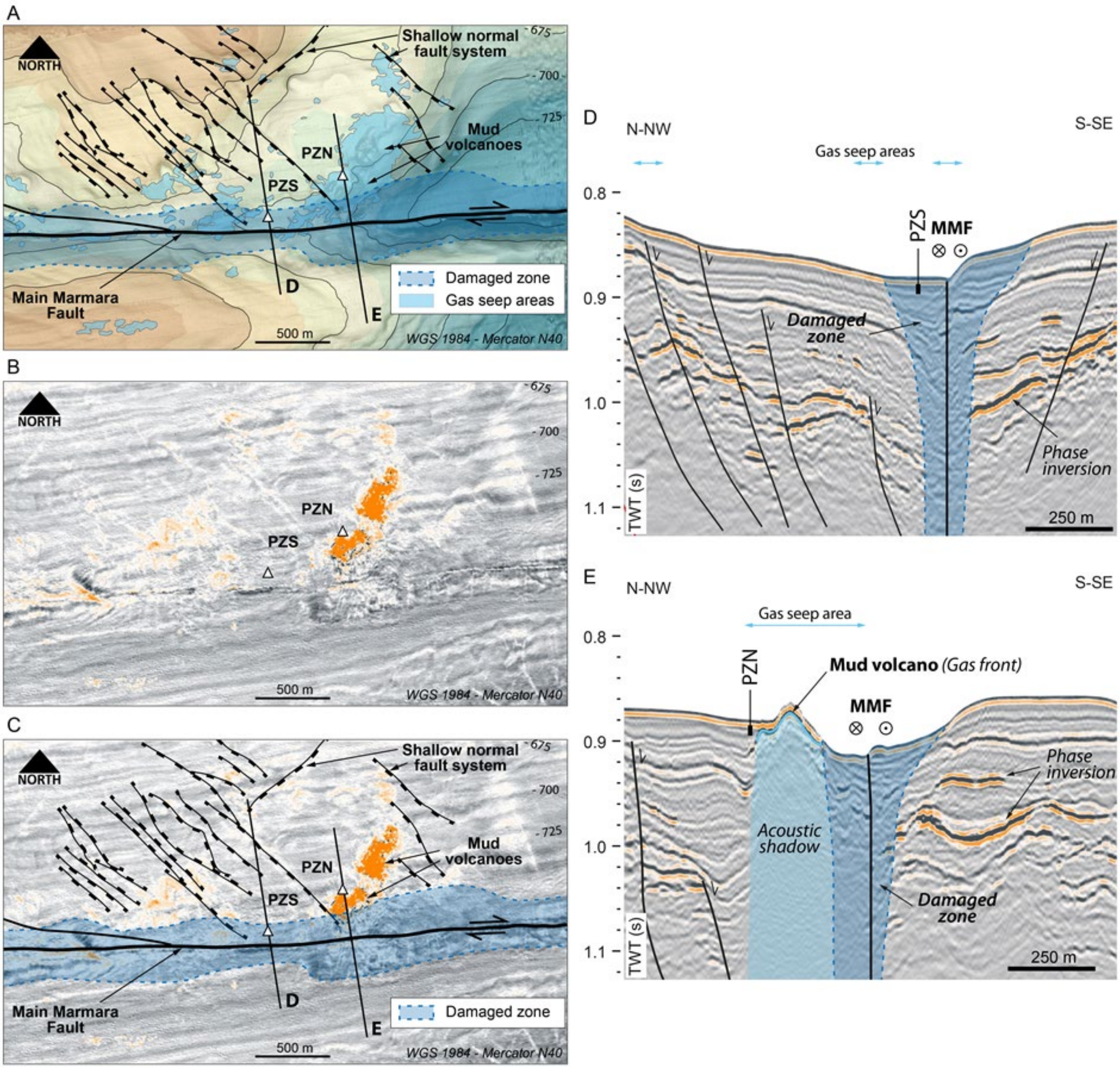

Supplementary Fig. 1. Seabed Amplitude and sub-seabed seismic features. *(a) General map showing the study area. Continuous black lines indicate the seismic lines shown in d & e. (b & c) The footprint of mud volcanoes (MVs) on the seabed is characterized by a high reflectivity while the trace of the Main Marmara Fault (MMF) is visible thanks to the discontinuity in the seabed amplitude derived from 3D seismic data. The damaged zone signature surrounding the MMF corresponds to a chaotic reflectivity surrounding the MMF. (d & e) Interpreted, high-resolution seismic lines indicating the position of piezometers PZN and PZS with respect to the MV, the MMF and its damaged zone.*

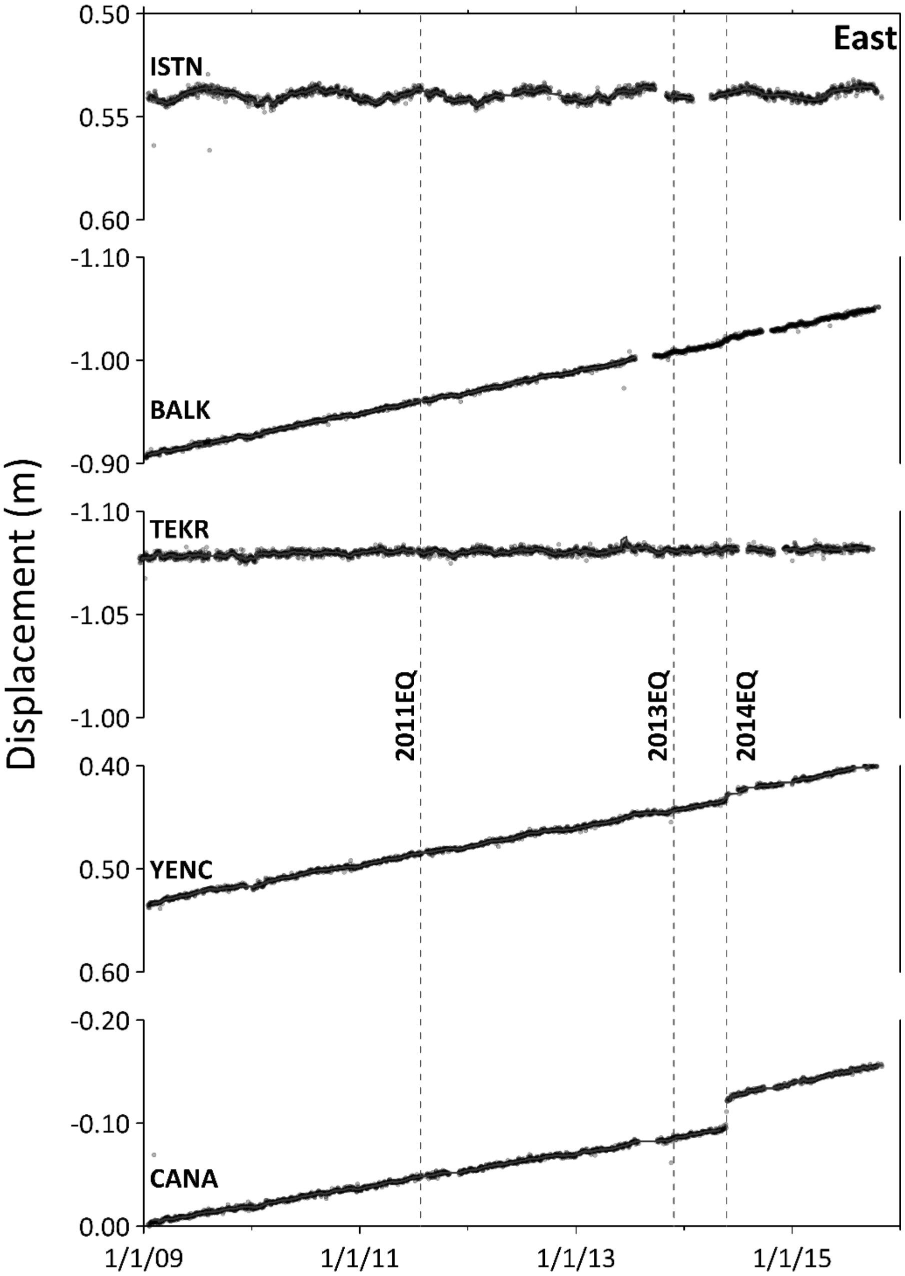

Supplementary Fig 2. Displacement time series (east). *Data from five selected GPS geodetic sites surrounding the Marmara sea (from west to east: CANA, YENC, TEKR, BALK and ISTN stations). Black dots and lines are the daily positions and running average fits. East displacement components are given in an EU plate fixed reference frame. The three vertical dashed lines indicate the occurrence of three neighboring earthquakes (called 2011EQ, 2013EQ and 2014EQ). The horizontal scale uses the convention of day, month, and year.*

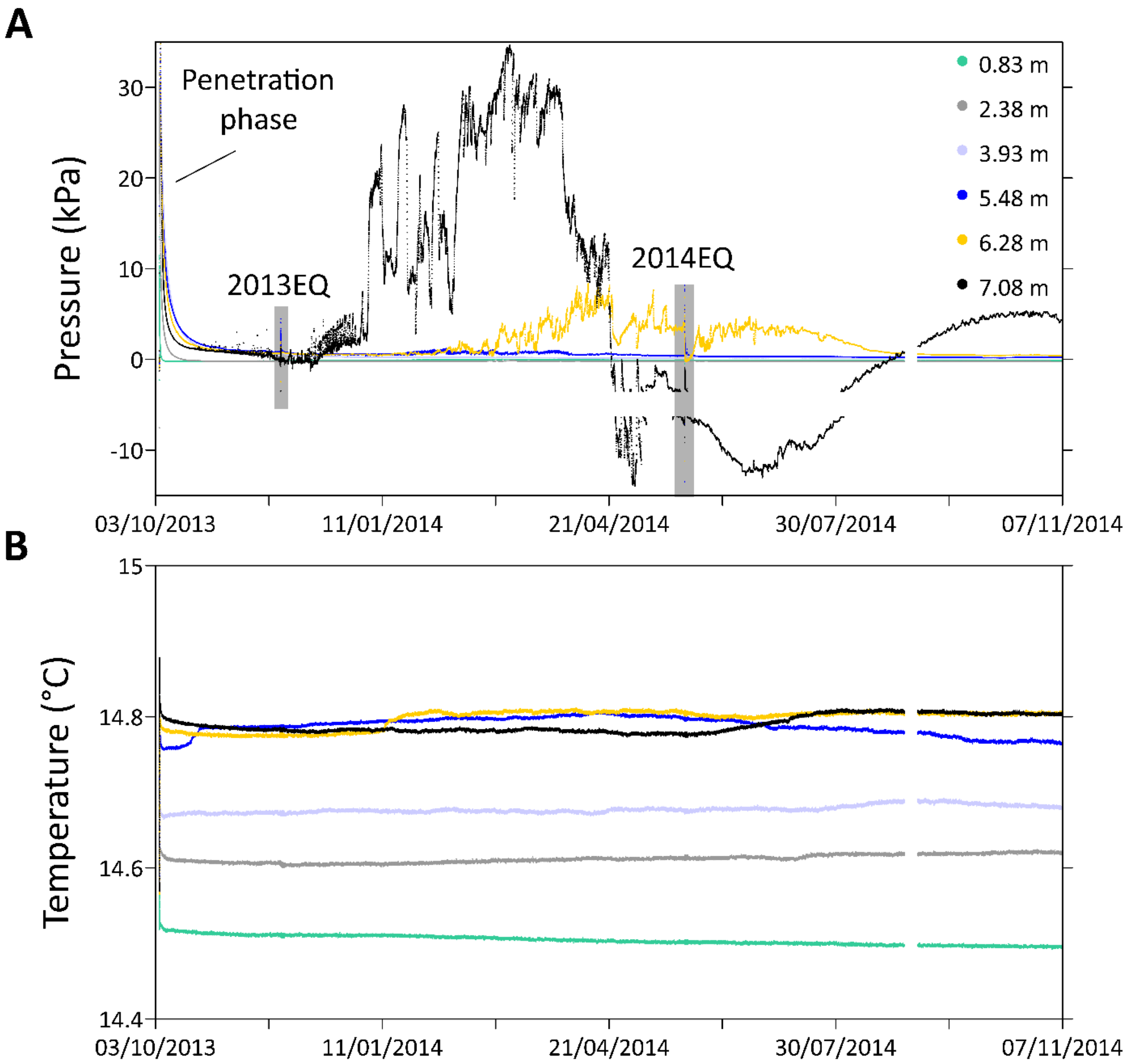

Supplementary Fig. 3. Data from piezometer site PZN. *(a) pore pressure and (b) temperature versus time. The different colors indicate the depth below the seabed. Sensor depths are between 0.83 mbsf (green curve) and 7.08 mbsf (black curve). The two earthquake events (2013EQ and 2014EQ) are indicated in (a). The horizontal scale uses the convention of day, month, and year.*

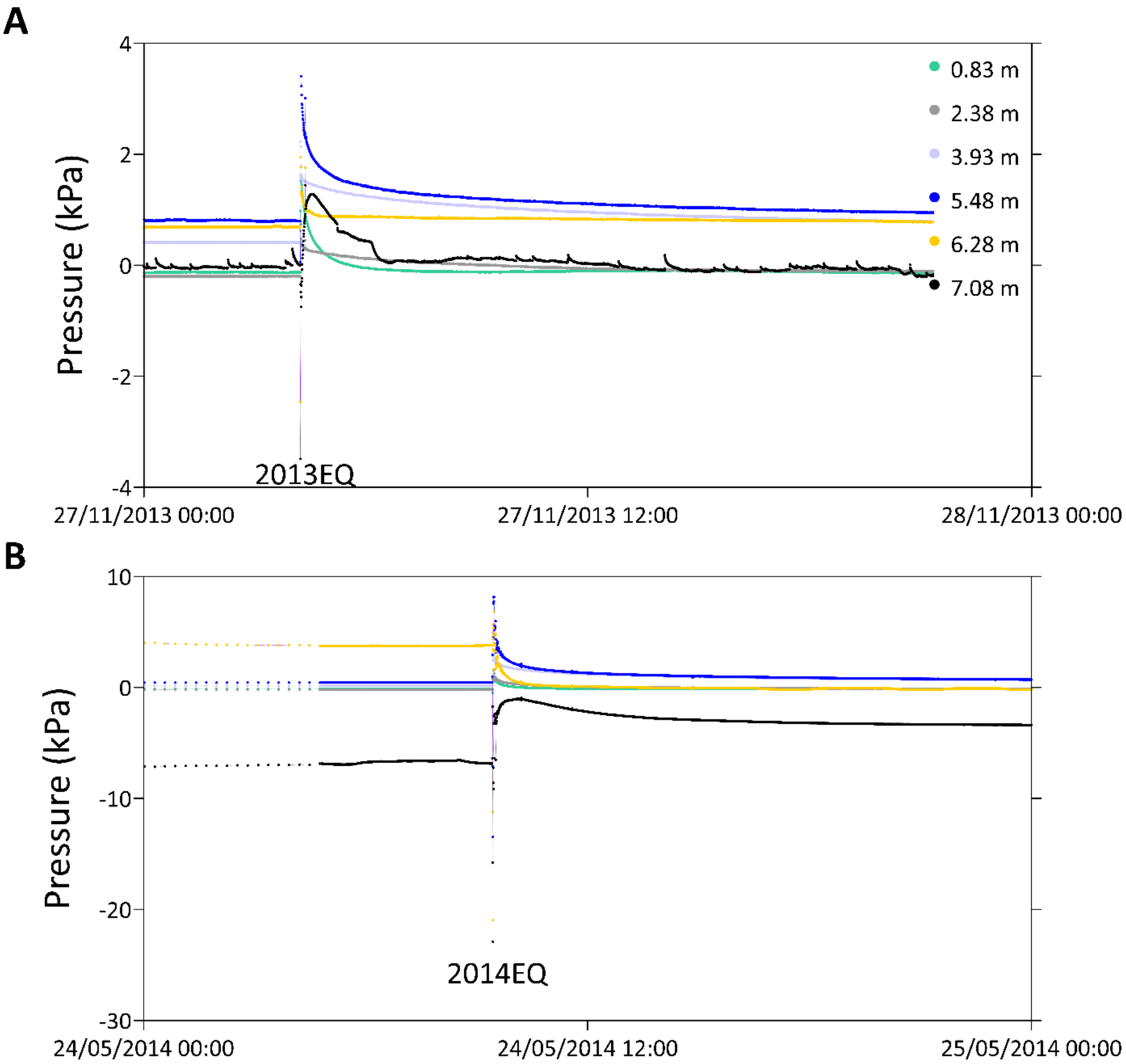

Supplementary Fig. 4. Enlargement of a part of data from piezometer site PZN. *Diagrams showing the pore pressure evolutions during (a) 2013EQ and (b) 2014EQ. The different colors indicate the depth below the seabed. Sensor depths are between 0.83 mbsf (green curve) and 7.08 mbsf (black curve).*

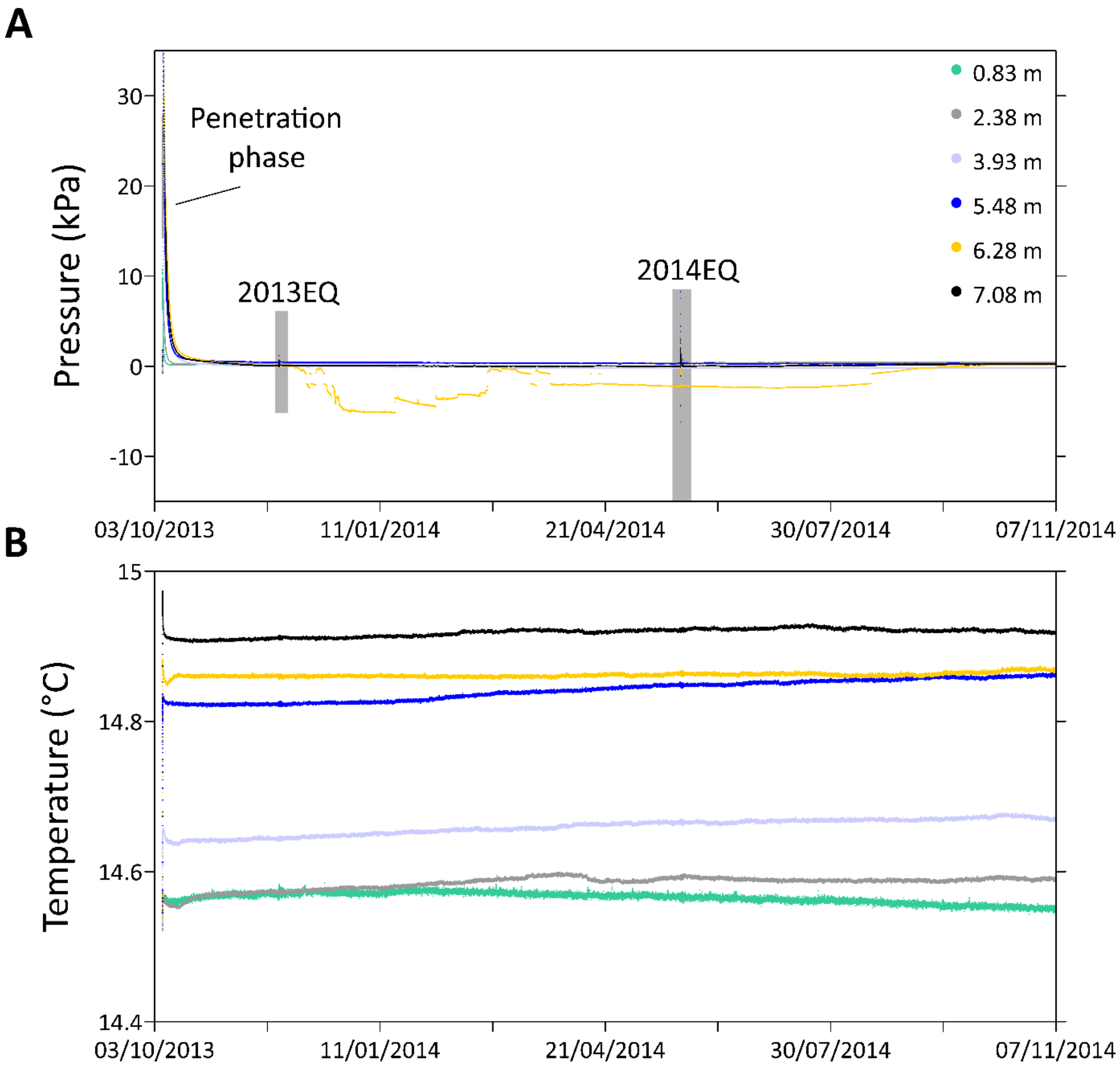

Supplementary Fig. 5. Data from piezometer site PZS. *(**a**) pore pressure and (**b**) temperature versus time. The different colors indicate the depth below the seabed. Sensor depths are between 0.83 mbsf (green curve) and 7.08 mbsf (black curve). The two earthquake events (called 2013EQ and 2014EQ) are shown in (**b**).*

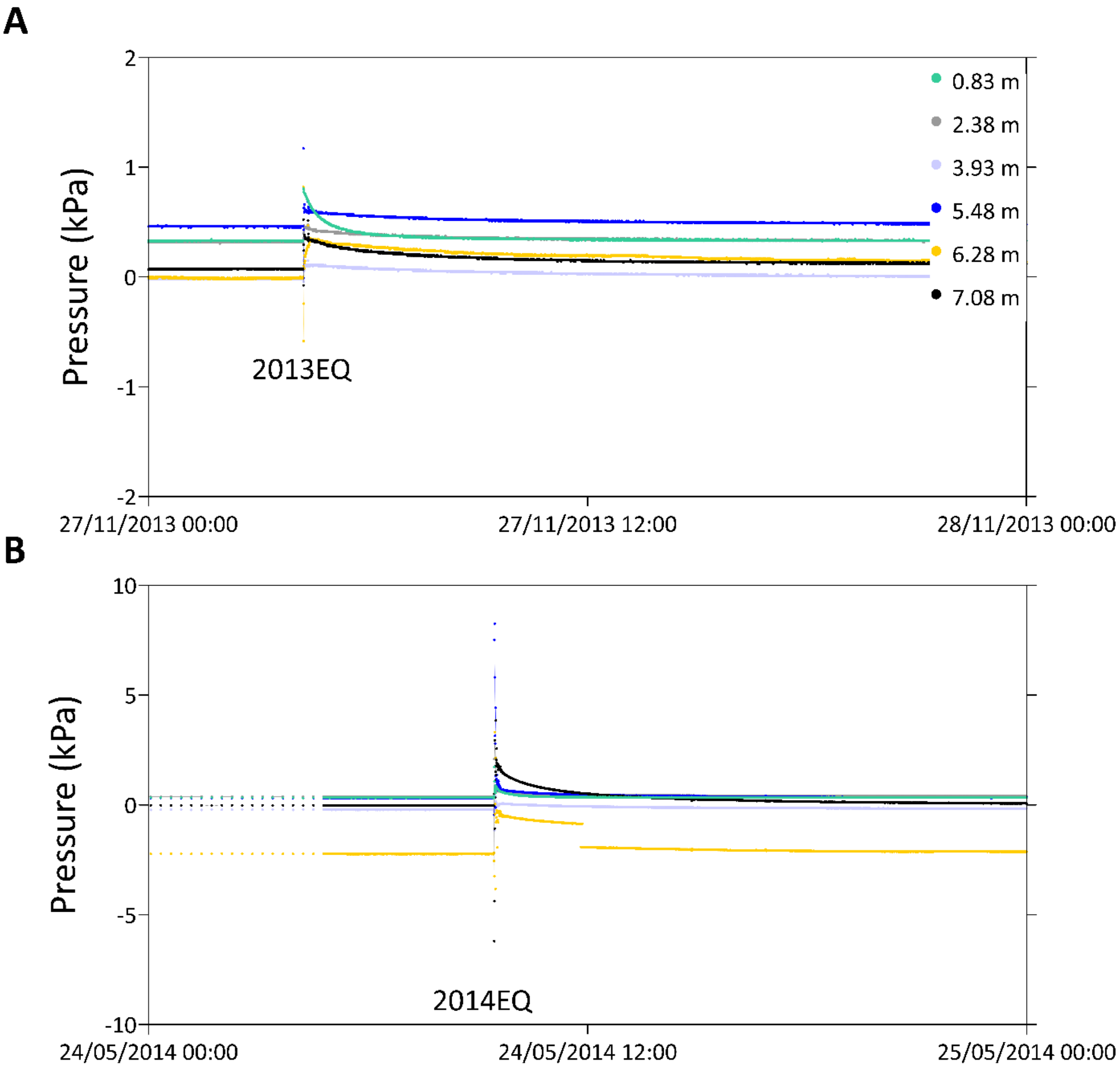

Supplementary Fig. 6. Enlargement of a part of data from piezometer site PZS. *Diagrams showing the pore pressure evolutions during (a) 2013EQ and (b) 2014EQ. The different colors indicate the depth below the seabed. Sensor depths are between 0.83 mbsf (green curve) and 7.08 mbsf (black curve).*

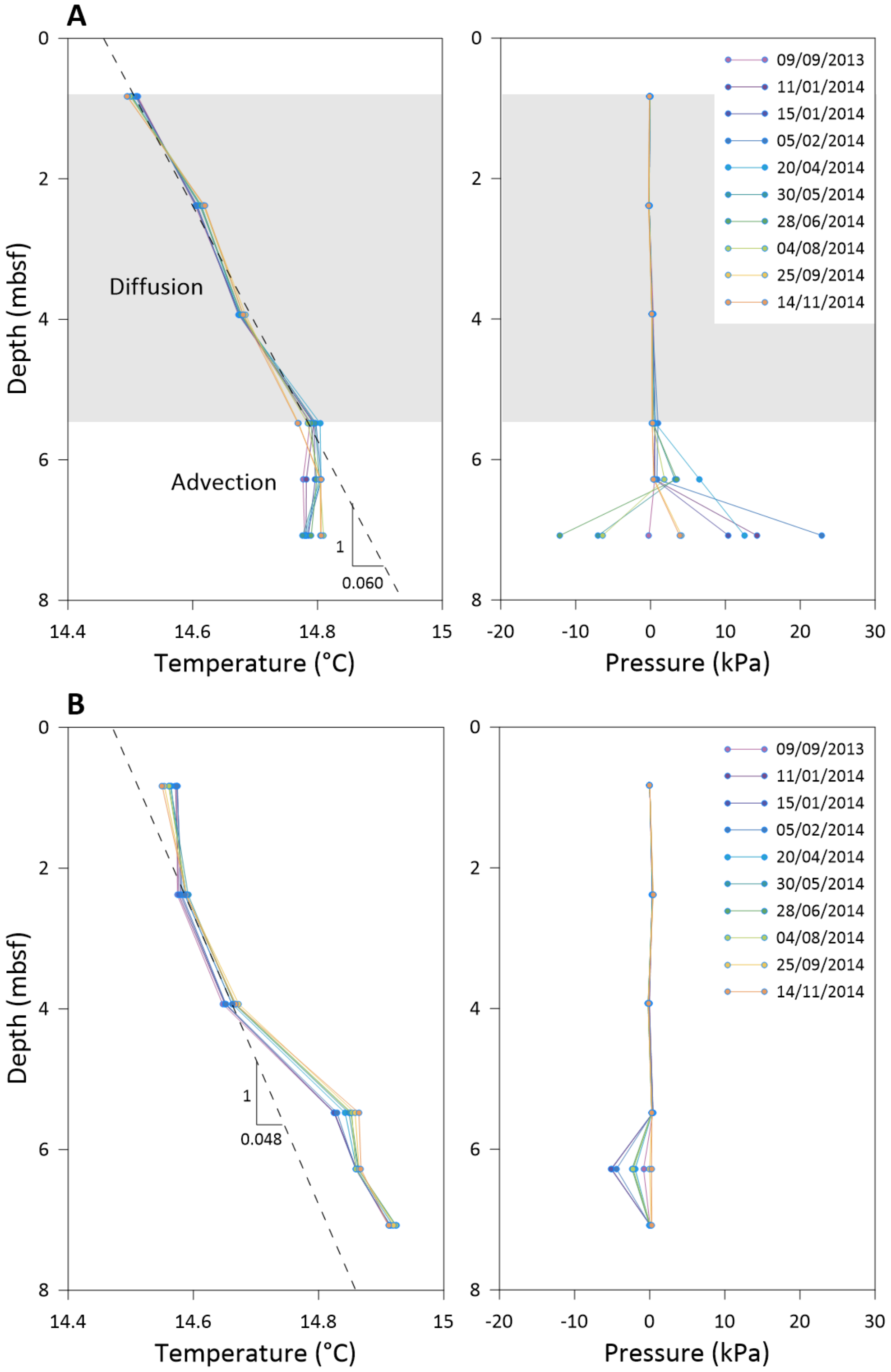

Supplementary Fig. 7. Thermal and hydraulic processes. *Thermal and pressure gradients at 10 different time periods derived from piezometer data. (a) PZN and (b) PZS.*

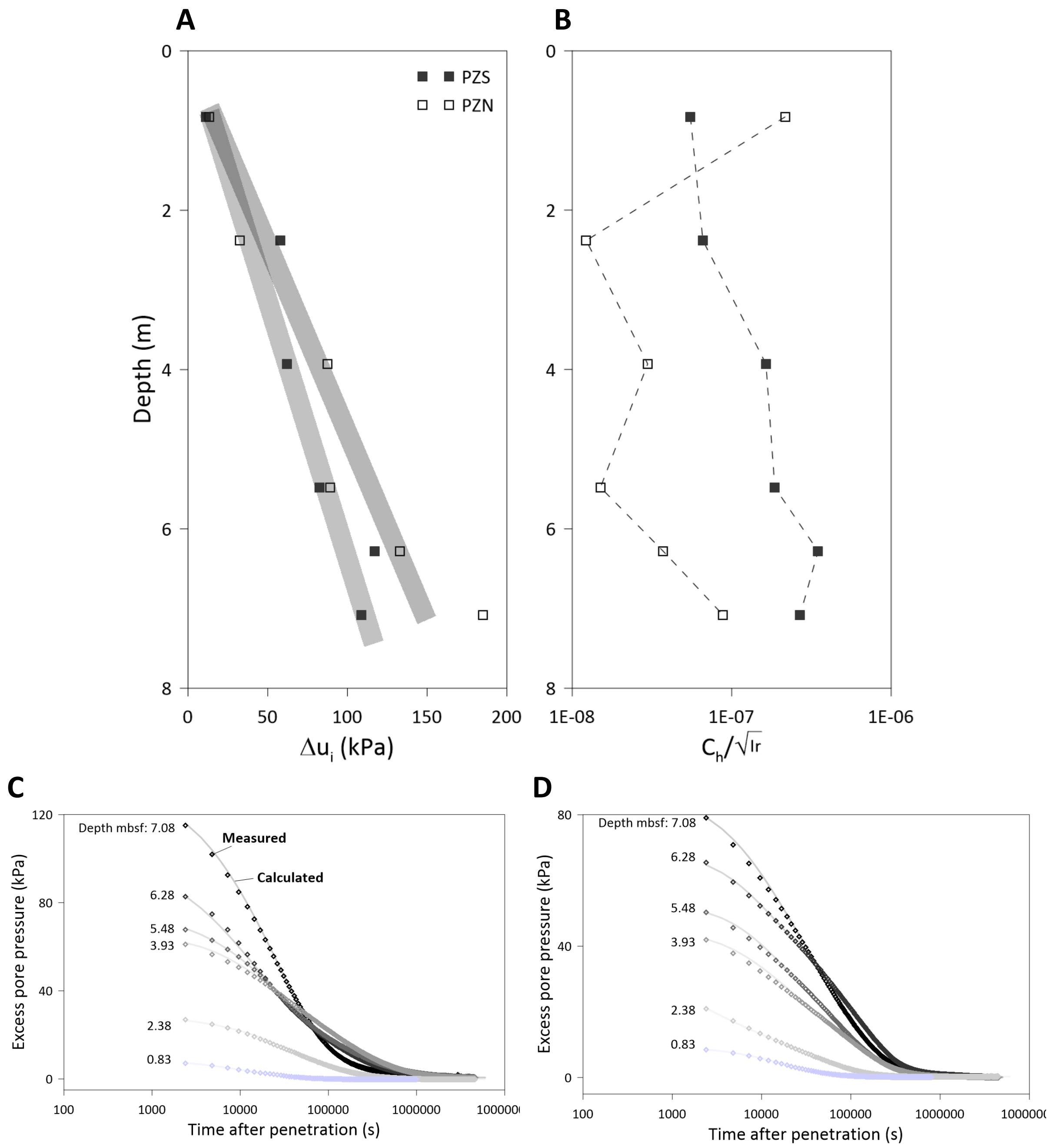

Supplementary Fig. 8. Hydro-mechanical properties of sediments at piezometer sensor positions. *(a) The initial excess pore pressure pulse (Δu_i) and (b) the horizontal coefficient of consolidation (C_h) normalized by square root of rigidity index ($\sqrt{Ir}$) as a function of depth. Measured (dots) and calculated (lines) pore pressure decay using the cavity expansion theory at piezometers PZN (c) and PZS (d).*

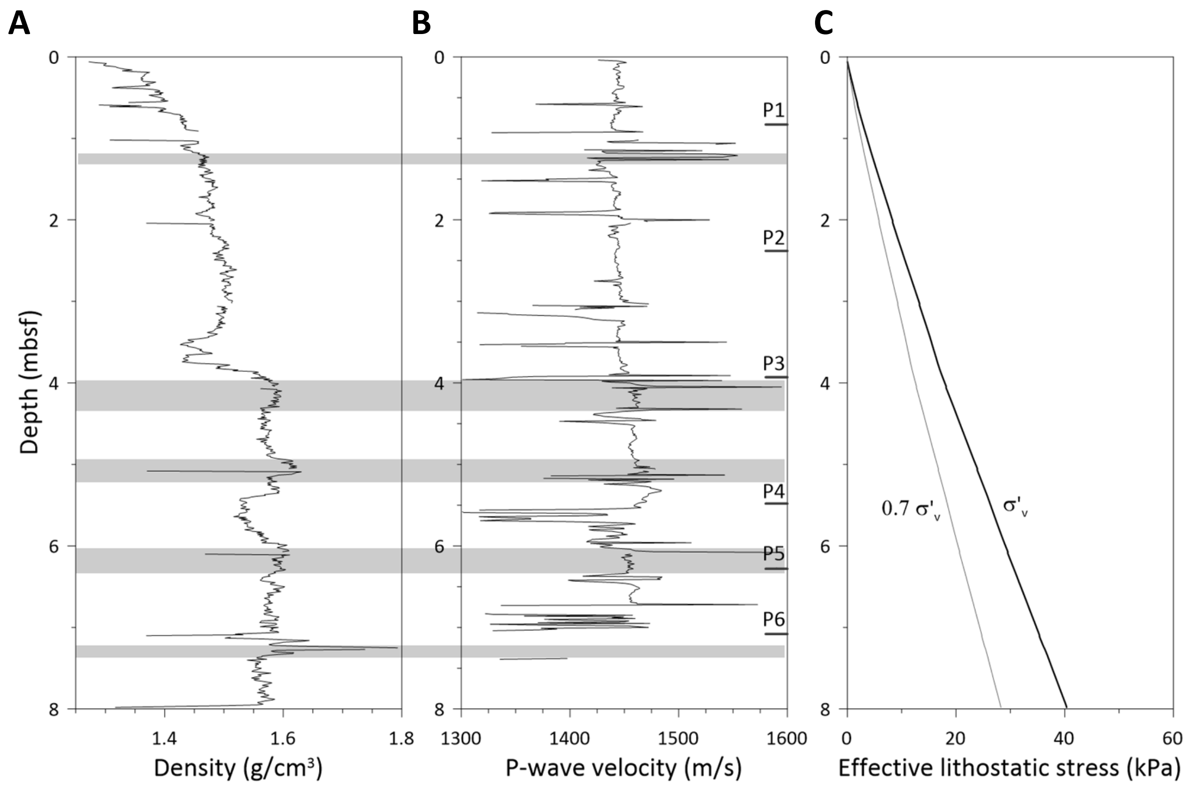

Supplementary Fig. 9. Core MNT-KS26. *(a) Density, (b) P-wave velocity and (**c**) effective lithostatic stress profiles (σ_v') from core MNT-KS26. Location of the core is shown in Fig. 1. High density and high P wave velocities (grey zones) possibly indicate the presence of sediment with high silt/sand content.*

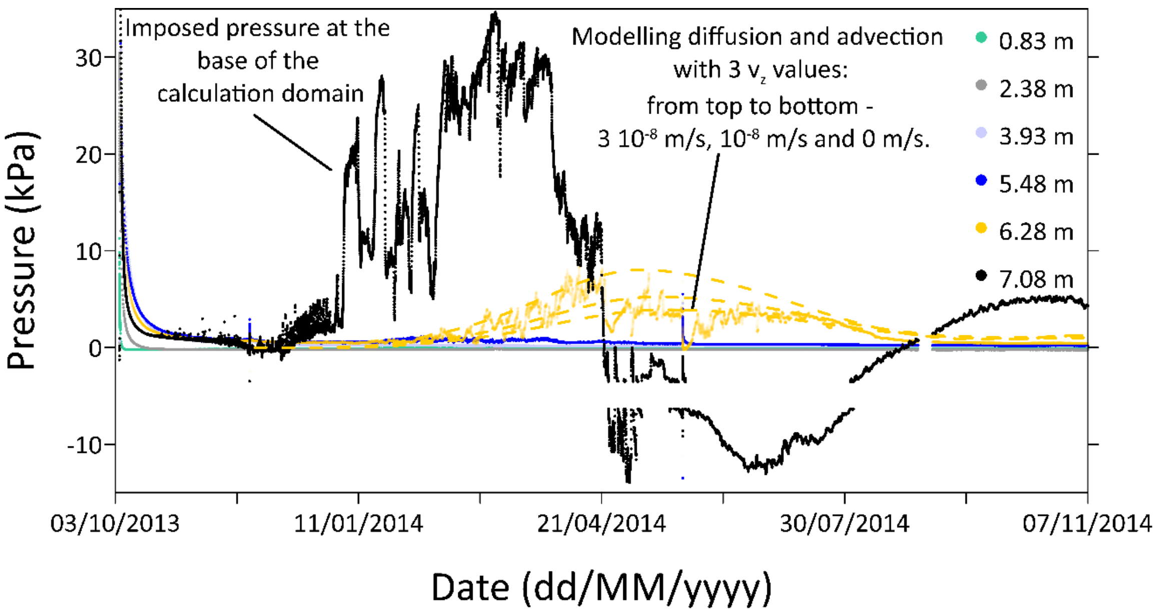

Supplementary Fig. 10. Transient diffusion-advection at piezometer site PZN. *Predicted pressure field in the calculation domain. The calculated pressure at 6.28 mbsf (yellow dashed curve) is compared to the measured one (continuous yellow curve). Depths are between 0.83 mbsf (green dots) and 7.08 mbsf (black dots).*

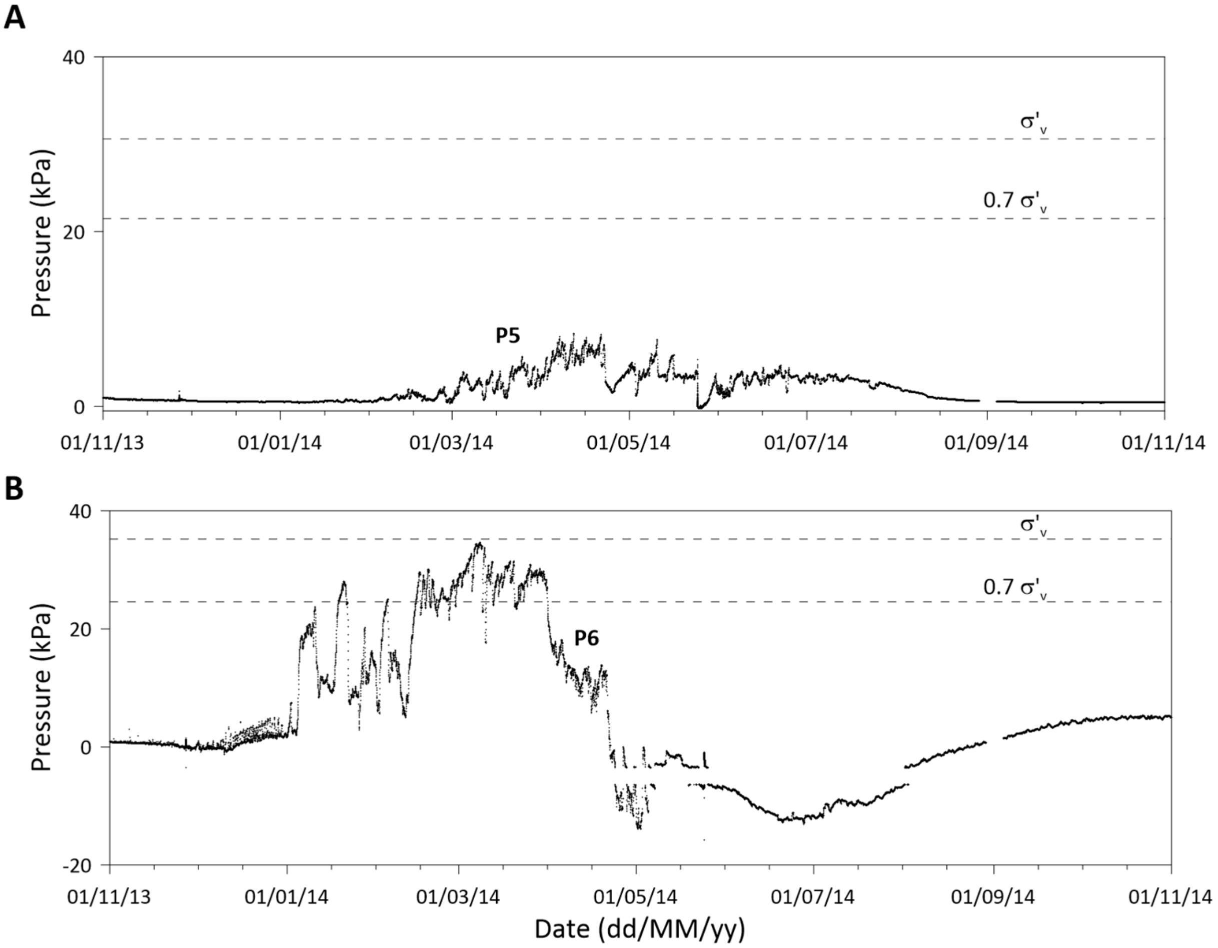

Supplementary Fig. 11. Hydro-fracturing at site PZN. *Pore pressure time series compared to effective lithostatic stresses (σ_v') at piezometers PZN-P5 (a) and PZN-P6 (b). PZN corresponds to the piezometer site and P5 and P6 to sensors at 6.28 and 7.08 mbsf (meter below seafloor).*

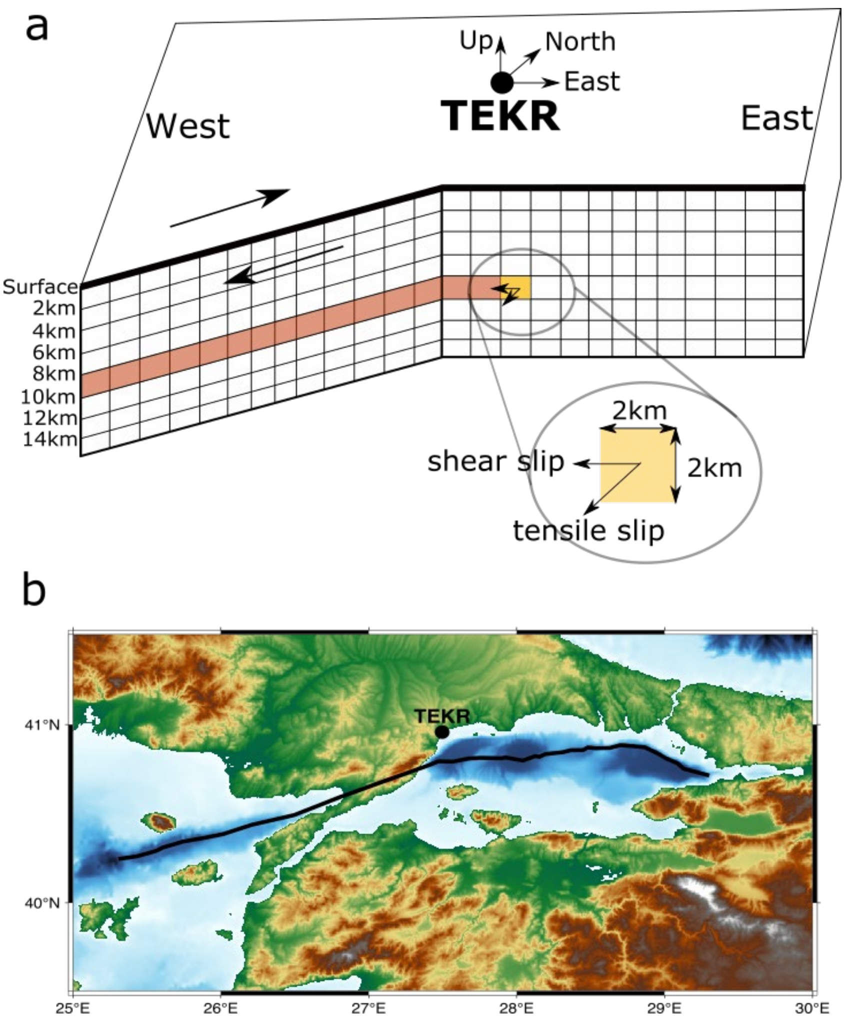

Supplementary Fig. 12. Schematic of fault discretization. A) *Thick black line represents the digitized fault trace with the large arrows indicating the predominant geodetic motion along the fault. Assuming the fault extends down vertically below the subsurface it has been subdivided into cells of 2km x 2km size along the trace. The black dot represents the geodetic station TEKR and the three components of displacement. The displacement at TEKR for 1m of right lateral slip and 1m of tensile for each cell on the fault has been calculated. These cells are summed sequentially for right lateral slip at a given depth – in the schematic an example is given for a slip propagating from west to east at a depth of 8-10km. The tensile slip is considered transitory and is not summed – in the schematic this represented by the yellow box which is considered to be the current active cell. B) Thick black line depicts the fault trace considered in simulation relative to TEKR (black dot). Earth relief provided by SRTM15+V2.1*[55]*.*

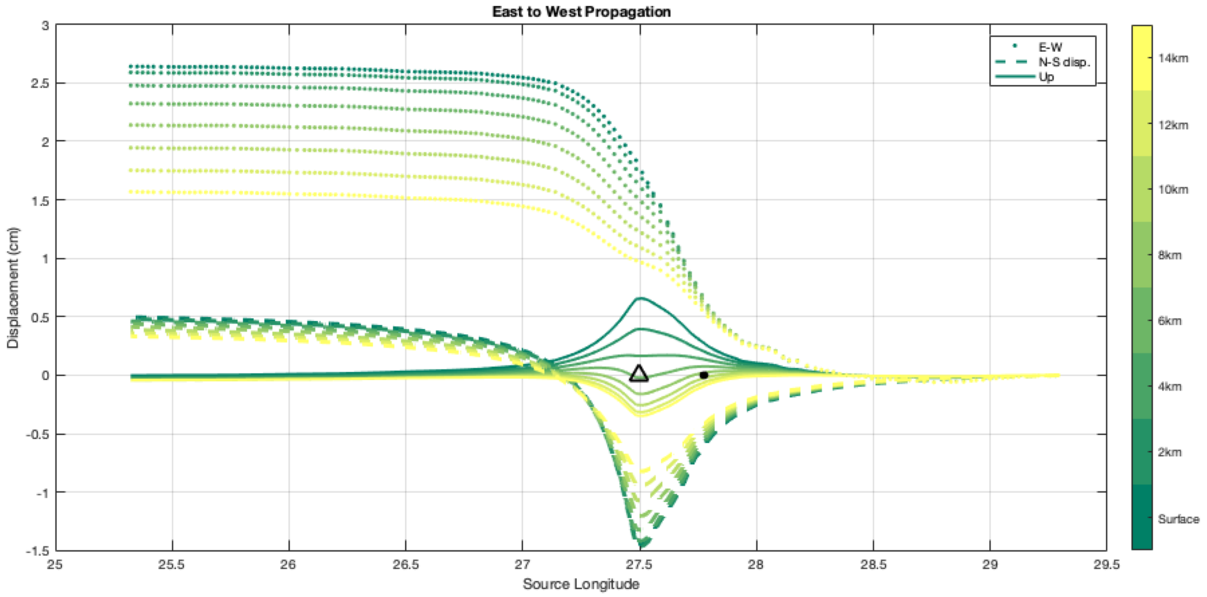

***Supplementary Fig. 13**. Case A: East to west propagation of a purely strike slipping front.* *The longitudinal barycenter of the actively slipping element is plotted along the x-axis. While on the y-axis, the solid lines represent the vertical displacement (positive up) and the dashed lines represent the north-south displacement (north direction is positive). The depths defined in the color bar represent the top of the slipping element. Black triangle represents location of the TEKR station and the black dot is the location of the PZS and PZN stations.*

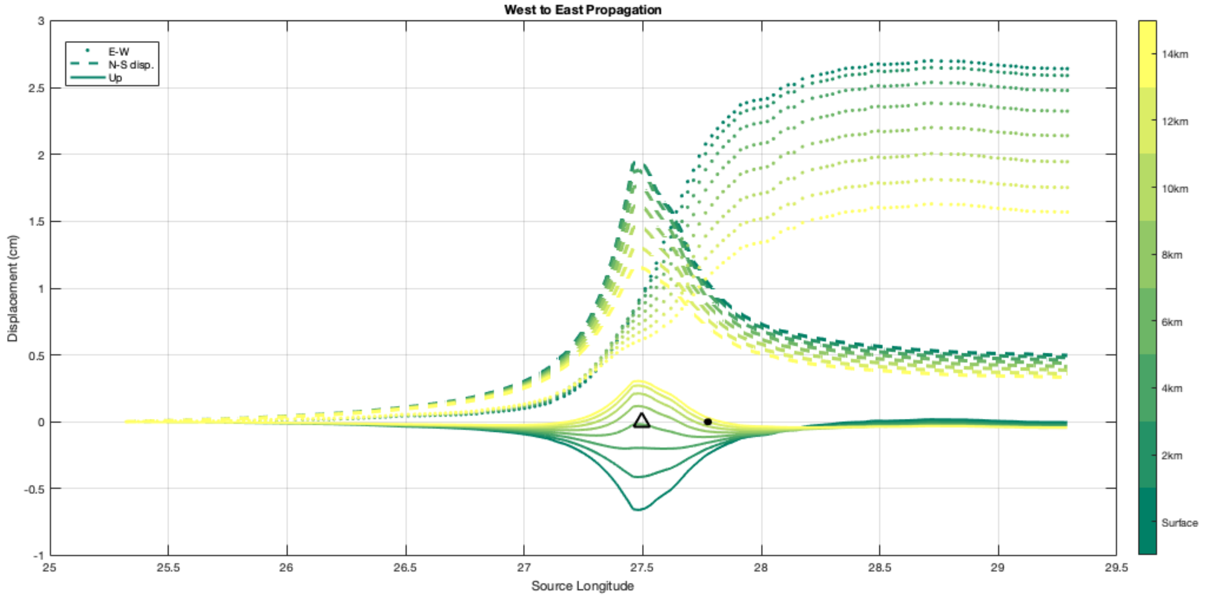

***Supplementary Fig. 14**. **Case B: West to east propagation of a purely right lateral slipping front.** The longitudinal barycenter of the actively slipping element is plotted along the x-axis. While on the y-axis, the solid lines represent the vertical displacement (positive up) and the dashed lines represent the north-south displacement (north direction is positive). The depths defined in the color bar represent the top of the slipping element. Black triangle represents location of the TEKR station and the black dot is the location of the PZS and PZN stations.*

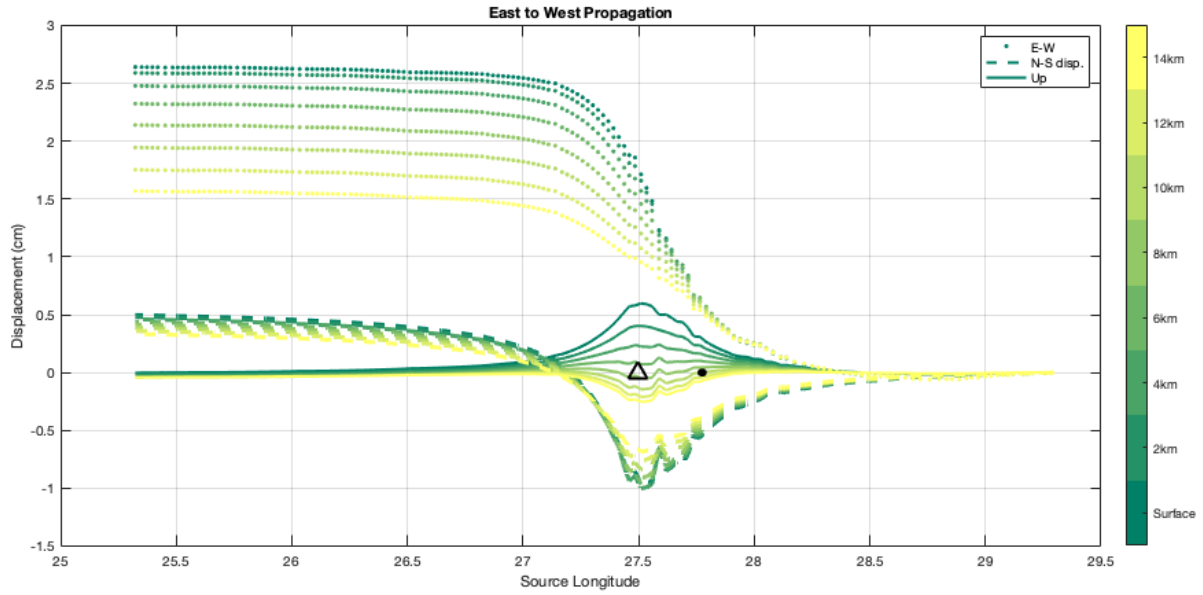

***Supplementary Fig. 15**. **Case C: East to west propagation of right lateral slipping front with a transient tensile component.** The longitudinal barycenter of the actively slipping element is plotted along the x-axis. While on the y-axis, the solid lines represent the vertical displacement (positive up) and the dashed lines represent the north-south displacement (north direction is positive). The depths defined in the color bar represent the top of the slipping element. Black triangle represents location of the TEKR station and the black dot is the location of the PZS and PZN stations.*

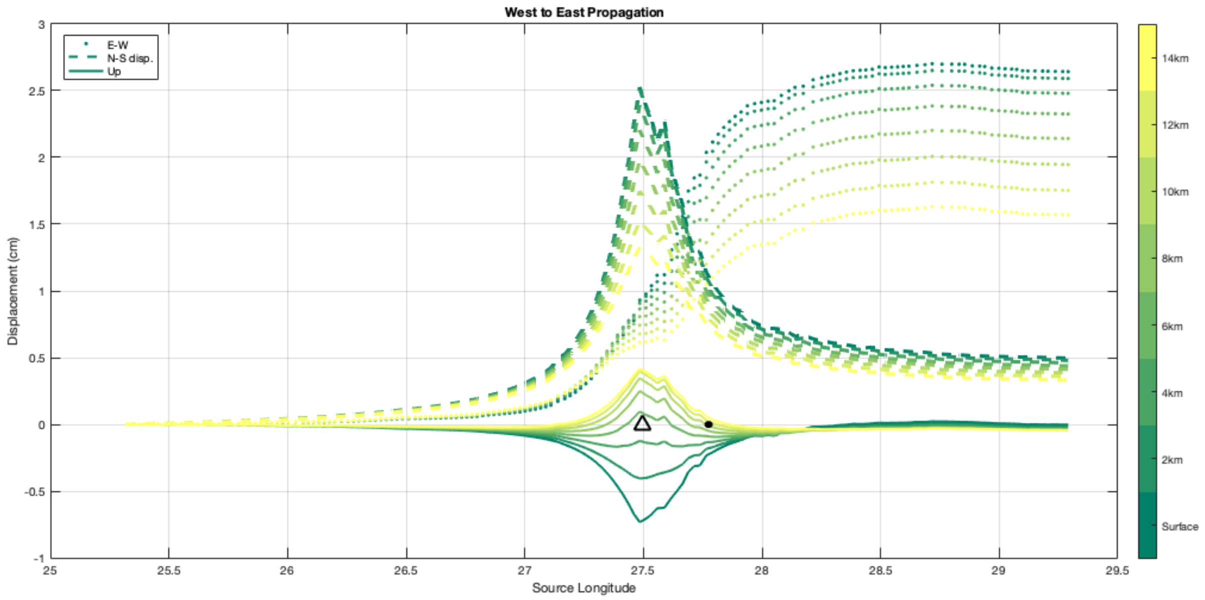

Supplementary Fig. 16. Case D: West to east propagation of right lateral slipping front with a transient tensile component. *The longitudinal barycenter of the actively slipping element is plotted along the x-axis. While on the y-axis, the solid lines represent the vertical displacement (positive up) and the dashed lines represent the north-south displacement (north direction is positive). The depths defined in the color bar represent the top of the slipping element. Black triangle represents location of the TEKR station and the black dot is the location of the PZS and PZN stations.*

Calculations	Right-lateral slip	Dilatancy	Depth of the imposed displacement	At TEKR – general tendency	
				Fitting with observed northern component	Fitting with observed vertical component
A	East to west	No	0-2 km<br>2-4 km<br>4-6 km<br>6-8 km<br>8-10 km<br>10-12 km<br>12-14 km	Y<br>Y<br>Y<br>N<br>N<br>N<br>N	Y<br>Y<br>Y<br>Y<br>Y<br>Y<br>Y
B	West to east	No	0-2 km<br>2-4 km<br>4-6 km<br>6-8 km<br>8-10 km<br>10-12 km<br>12-14 km	N<br>N<br>N<br>Y<br>Y<br>Y<br>Y	N<br>N<br>N<br>N<br>N<br>N<br>N
C	East to west	Yes	0-2 km<br>2-4 km<br>4-6 km<br>6-8 km<br>8-10 km<br>10-12 km<br>12-14 km	Y<br>Y<br>Y<br>Y<br>N<br>N<br>N	Y<br>Y<br>Y<br>Y<br>Y<br>Y<br>Y
D	West to east	Yes	0-2 km<br>2-4 km<br>4-6 km<br>6-8 km<br>8-10 km<br>10-12 km<br>12-14 km	N<br>N<br>N<br>Y<br>Y<br>Y<br>Y	N<br>N<br>N<br>N<br>N<br>N<br>N

Supplementary Table 1. Summary of results from simulations. *Best-fit cases are indicated by blue text.*
